# Supplementary material for: Optimization of a WGA-Free Molecular Tagging-Based NGS Protocol for CTCs Mutational Profiling
Source: Int J Mol Sci. 2020 Jun 19;21(12):4364. doi: 10.3390/ijms21124364 (PMC7352435; doi:10.3390/ijms21124364)
Supplement: Supplementary file 1 [file ijms-21-04364-s001.pdf]

Table S1. Details of single NGS library preparation steps in both canonical and optimized workflows

	Canonical	Optimized
1st amplification step (Molecule tagging) components:		
Breast cfDNA Panel v2	2µl	0.6µl
cfDNA Library PCR Master Mix	15µl	5µl
Nuclease-free water	0µl	1.4µl
DNA input	10µl normal genomic DNA (5ng) plus 3µl DNA from MDA sorted cells on DEPArray	3µl DNA from MDA sorted cells on DEPArray
**Total volume**	**30µl**	**10µl**
1st purification and elution steps:		
Agencourt Ampure XP Reagent beads	45µl	15µl
80% ethanol	150µl	100µl
Low TE	24µl	8µl
2nd amplification step (Library generation) components:		
DNA from 1st elution step	23µl	8µl
Tag Sequencing BC (1-24)	1µl	0.4µl
cfDNA Library Primer P1	1µl	0.4µl
cfDNA Library PCR Master Mix	25µl	8µl
**Total volume**	**50µl**	**16.8µl**
2nd purification and elution steps:		
Agencourt Ampure XP Reagent beads	57.5µl	19.3µl
80% ethanol	150µl	100µl
Low TE	50µl	16.8µl
Size selection step:		
DNA from 2nd elution step	50µl	16.8µl
Agencourt Ampure XP Reagent beads	50µl	16.8µl
80% ethanol	150µl	100µl
Low TE	30µl	22µl
**Final transferred library volume**	28µl	20µl
